# Gelation of Textile Dye Solution Treated with Fish Scales

**DOI:** 10.3390/gels5030037

**Published:** 2019-07-18

**Authors:** S M Fijul Kabir, Taslim Ur Rashid, Ioan I. Negulescu

**Affiliations:** 1 Department of Textile Engineering, Chemistry and Science, Wilson College of Textiles, North Carolina State University, Raleigh, NC 27695, USA; 2 Department of Textiles, Apparel Design and Merchandising, Louisiana State University, Baton Rouge, LA 70803, USA

**Keywords:** fish scales, dye solution, gel, rheology

## Abstract

In the present article, the commercial value of fish scales (FS), one of the most discarded fish wastes, has been identified by discovering their gelation capability. Fish scales of different physical forms were applied for the removal of dyes (acid red 1 (AR1), acid blue 45 (AB45), and acid yellow 127 (AY127)) from textile dye solution by absorption process. An astounding phenomenon, gelation of the treated solution, was noticed when it was aged for a certain period. The absorption of dye by FS was confirmed and quantified by FT-IR and UV-visible spectroscopy analyses, respectively. Process optimization revealed that pristine FS showed better gelation efficacy compared to pulverized FS. The gelation process was successful only when the dye solution contained acid and salt. As most of the textile effluents contain acids and salts in the discarded dye solution, this gelation process implies an obvious indication of the saving process and chemical cost in textile waste treatment. The jellified wastewater was characterized by exploring the rheological properties. Based on these analyses, potential application areas have been discussed.

## 1. Introduction

Fish wastes, including heads, bones, skin, scales, fins, and viscera, could account for up to three fourths of the total fish weight and are dumped carelessly into land or sea, resulting in a negative environmental impact [1]. These wastes are generated as a byproduct during the fillet processing in the fish industries. Arvanitoyannis and Cassavetes [2] mentioned the amount of scales from fish processing can range from 20 to 40kg per 1000kg of fishes during filleting. Although fish scales (FS) could be used as a potential bio-sorbent for metals and dyes [3,4,5], an alternative source of collagen, chitin or chitosan extraction [6,7,8,9] as well as for ornamentation purpose [10,11], there are few commercial applications. 

FS contain 40%–90% organic protein matters including type-I collagen composed of a high amount of proline, glycine, alanine, hydroxylysine, hydroxyproline, and 10%–60% inorganic mineral, calcium-deficient hydroxyapatite composed of sodium, magnesium, and carbonates attached to phosphate of the hydroxyapatite ions regardless the types of fish [12,13]. The thermal denaturation of collagen forms gel, which might occur due to leaching protein content during the absorption process investigated in the study. Particularly, the gelation in this process might be very significant, since the traditional major source of gelling agent practiced by the food industries includes gelatin, which is mainly manufactured from pig skins, cattle hides, and pig and cattle bones, and most of these are considered restricted items for some religions such as Judaism, Hinduism, and Islam [1]. 

In the present study, the gelation, an additional and unexpected finding observed in the dye absorption process, was obtained following a very simplistic process with few steps and involved no remarkable physical or chemical modification of FS. Finally, the rheological behavior of the gel has been explored to understand the nature of the gel. In addition, an attempt has been made to explain the chemistry and major governing factors of the gelation process. 

## 2. Results and Discussion

### 2.1. Absorption of the Dye

The fish scales showed almost similar dye removal and gelation performance for all types of dye. For simplicity of reporting, the characterization techniques will be described for the case of AB45 dye solution only.

The FS, collected after treating the bath solution containing AB45 dye, was found in the blue color. Another significant observation was that the dried fish scales became brittle after dye absorption, while they were ductile before treatment. One of the probable reasons of brittleness was leaching and hydrolysis of proteins and minerals from FS during dye solution treatment. 

#### 2.1.1. FT-IR Analysis

**Figure 1:**
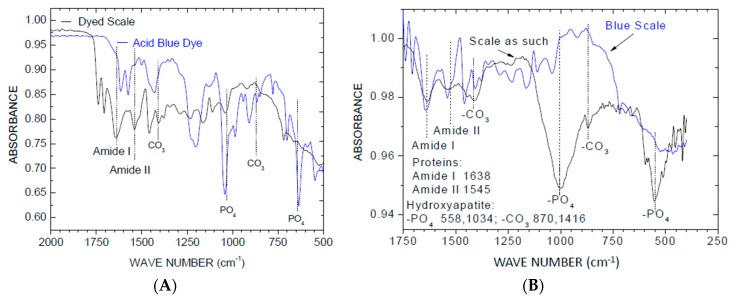
FTIR spectra of the blue colored fish scale and the AB45 dye used for scale coloration (**A**), and the pristine (as such) fish scales (FS) and of the blue colored scale (**B**).

The Fourier-transform infrared spectra (FT-IR) of the AB45 dye, pristine FS and dyed scales showed the absorption characteristic peaks for the functional groups of dye, peptide (amide) groups of collagen in FS, -NH-C(=O)-, labelled as Amide A, Amide I, and Amide II, together with the bands typical to absorbances for phosphate and carbonate ions in the apatite lattice containing hydroxyapatite, Ca_10_(PO_4_)_6_(OH)_2_, and calcium carbonate, CaCO_3_, are presented in Figure 1A,B [14,15]. The presence of characteristic peaks for the functional groups of AB45 in dyed FS confirmed the absorption of dye by the scales.

#### 2.1.2. Dye Removal Efficiency

The concentration of dye in the raw and treated solution was determined by UV-visible spectroscopy to calculate dye removal percentage and maximum dye absorption. The dye removal percentages for AB45 was around 89% and maximum dye absorption in FS 2.7 mg/g. Marrakchi et al. found a similar trend while examining those effects on reactive orange 16 and methylene blue dye removal by carbonized FS [16,17].

### 2.2. Characterization of Gel

#### 2.2.1. Nature and Chemistry of Gel Formation

The ageing of the treated dye solution left gel specially when it was treated with pristine FS and large pulverized FS. Several previous reports demonstrate that FS contained a significant amount of collagen [18]. We assume that when FS is treated with dye solution at an elevated temperature, the dye molecules are absorbed in active sites of FS. Simultaneously, the acid and salt content in the dye solution facilitates the acid hydrolysis of collagen releasing gelling agent, which helps gelation of the solution. As a result, FS are dyed due to the absorption of dye molecules, on the other hand, the residual dye solution turns into an almost transparent gel. This amazing dual function of FS opens a new avenue in the commercial application of the FS waste. The gel content, which is mainly the hydrolyzed product of collagen, was determined as 0.45% by calculating the difference between the percentage of solids obtained by evaporation (drying) of the liquids containing sodium sulfate, sulfuric acid, and dye before and after adding the scales for absorption process.

#### 2.2.2. Rheological Behavior of the Gel

**Figure 2:**
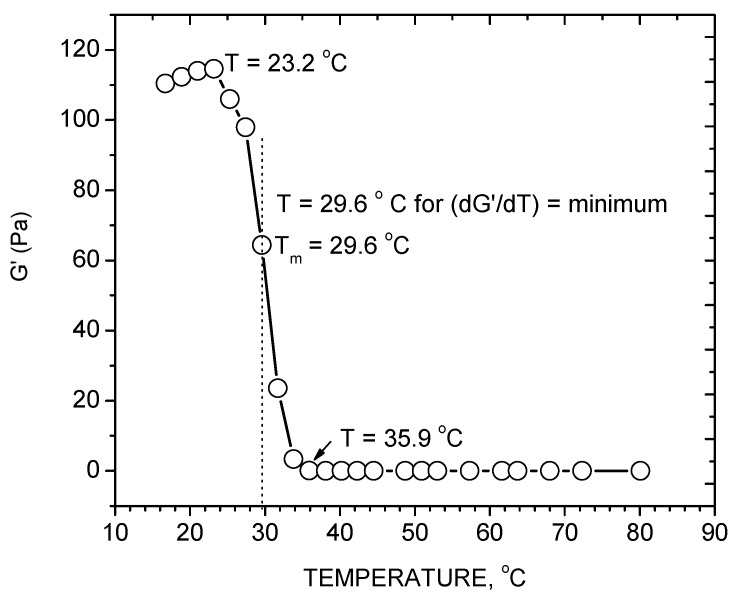
Rheological test for elastic modulus (G′) of the gel obtained from treated dye solution (AB45) upon heating. The curve represents the variation of G′ of the gel with temperature increasing from 15 to 80 °C.

**Figure 3:**
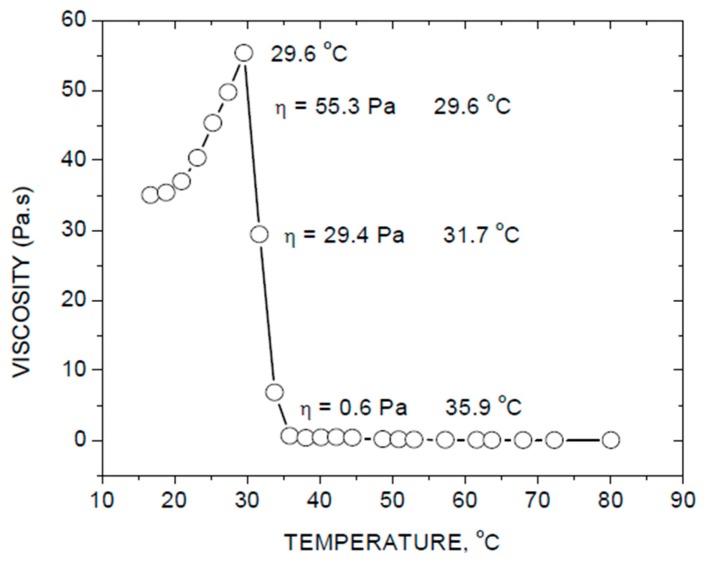
Rheological test for viscosity (η) of the gel obtained from the treated dye solution (AB45) upon heating. The curve represents the variation of η of the gel with temperature increasing from 15 to 80 °C.

The rheological measurements of the gel give an idea about the strength and thermal stability of the gel. The values of the elastic component (G’) and the viscosity (η) of the gel with temperature sweeping were directly obtained from the curve generated by the rheometer. The results showed that both the elastic component (G’) and the viscosity increased first with temperature when the system became non-homogeneous in the initial stages of melting, with a rapid collapse after gel melting at around 30 °C (Figure 2 and Figure 3, respectively), which evidenced that the gel strength was higher than the gel obtained from the fish wastes (bones and skins) of the tiger toothed croaker, pink perch, sea bass, nile tilapia, and red tilapia fishes [19,20]. Moreover, the melting point (30 °C) of the gel in the present study was notably high compared to that of other gels directly obtained from the fish scales (20.8 °C) and skins (26.9 °C) of the different fish source [21].

**Figure 4:**
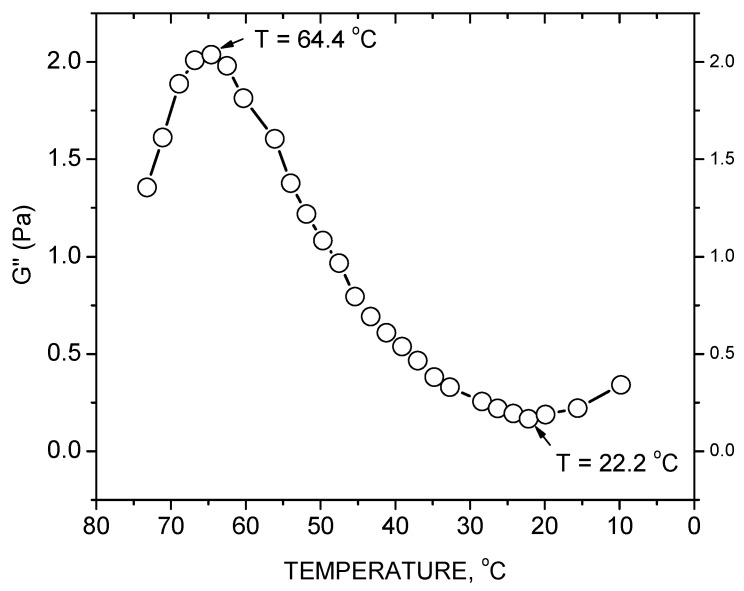
Rheological test for loss modulus (G″) of the molten gel upon cooling. The curve represents the variation of G″ of the molten gel with cooling from 75 to 10 °C.

**Figure 5:**
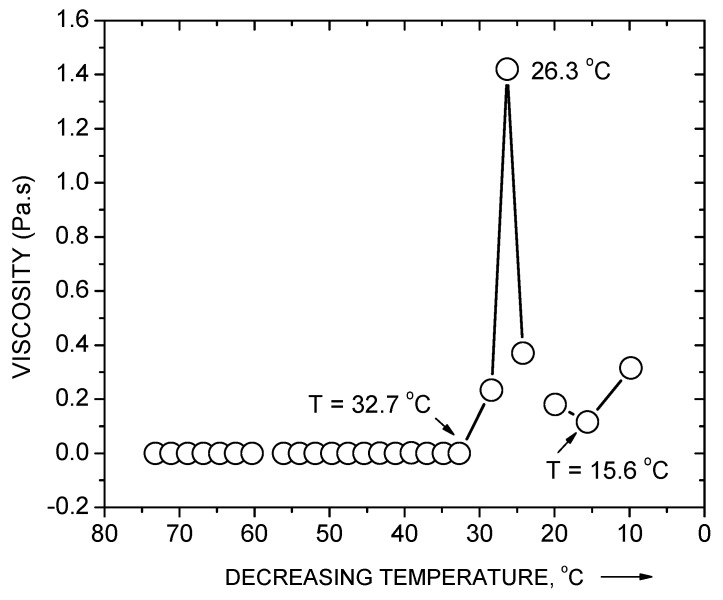
Rheological test for viscosity (η) of the molten gel upon cooling. The curve represents the variation of η of the molten gel with cooling from 75 to 10 °C.

A reverse scenario was noticed by cooling the collagen solution back to room temperature, in which G’ of the collagen solution increased as the temperature went down to 60 °C, decreased due to the non-homogeneity of the system in the temperature range preceding total jellification (around 30 °C again), and increased after in a normal way, i.e., G’ of a homogeneous system increases as temperature decreases. This irreversible nature of the gels indicated that it was a physical gel and the microstructure and strength of the gel were largely affected with change in the thermal profile of the system. It was also noticeable that the range of G’ varied from a firm gel (>100 Pa) to a liquid (Figure 2) and vice-versa to a weak gel (<10 Pa), as presented in Figure 4, which were much higher, as found by Koli et al. [19] for the gels extracted from the tilapia fish wastes. A similar scale reduction was observed for the viscosity of the gel from >50 Pads before melting (Figure 3, heating run) to <1 Pads of the week gel formed in the cooling run (Figure 5).

### 2.3. Factors Affecting the Gel Formation

#### 2.3.1. Size of Fish Scales

FS size showed a significant effect on the gel formation capacity. The gel formation was noticed when the treated wastewater solution was allowed to age for 24 h at room temperature, especially for pristine and large pulverized scales. No gelation was observed for the reduced size of the scales (mesh no. 40, 60, 100, and 200). It can be assumed that the proteins of the FS denatured during the size reduction process. Hence, protein hydrolysis was hampered during the dye removal process and there was no evidence of gelation. Another potential reason of such unusual behavior is that the size reduction of FS increases the active sites with increasing surface area, which helps rapid absorption of dye and salts instead of dissolution of proteins from FS to dye solution.

#### 2.3.2. Composition of Dye Solution

Although the dye absorption capacity of the FS decreases with increasing acid and salt content in the dye solution but the gelation capability increases. The presence of acid and salt increase the hydrolysis of proteins, which ultimately enhance the gelation process. This phenomenon added the extra advantage of the application of FS in textile effluent treatments as most of the textile effluent contains acids and salts along with dyes.

## 3. Application and Future Research Directions

The jellified proteins (collagen) obtained from different sources are processed as thin films, which might find some promising applications in medical and biomedical (protection, drug delivery), cosmetic (micro- and nano-scale protein ointments), and food (micro packaging) industries [22,23]. An application has been proposed and experienced to formulate Panna Cotta products using gelatin from tilapia wastes by Tinrat and Sila-asna [20]. Koli et al. described several methods of extracting the gelatin from fish skin and wastes. The way of obtaining gelatin described in the present study is easier in terms of time, chemicals, and process convenience; therefore, it could be a very promising method for future studies [19]. Nonetheless, this gel cannot be used in food processing directly due to the presence of hazardous materials like acids, salts, and residual dyes. The primary advantage of this gelation process is that it can reduce the liquid effluent disposal from the textile industry. The gel can be dried and disposed of properly as a solid waste, which will reduce the overall volume of the waste, as well as improve the ecofriendly waste management system. Moreover, future studies can be conducted to find out any possible way to process the gel to recycle the acid and salts to use as a nutrient carrier for plants in the agricultural field or to apply the dried gel as a dye or heavy metal absorbent from industrial effluents [23]. The colored FS could be used for many functional, ornamental and design applications [10]. 

## 4. Conclusions

In the present study, an amazing observation is reported that describes gelation of a dye solution when treated with fish scales and aged for at least 24 h while the dye molecules are absorbed by the FS. The suitability of fish scales (FS) as a prospective gelling agent was assessed by different chemical and instrumental analyses. Process performance was optimized by investigating different process variables such as dye composition, FS size etc. Future studies can be conducted to investigate the underlaying detailed chemistry of gel formation and properties of the jellified treated solution to find out potential applications of colored fish scales and gel.

## 5. Materials and Method

### 5.1. Materials and Chemicals

**Scheme 1:**
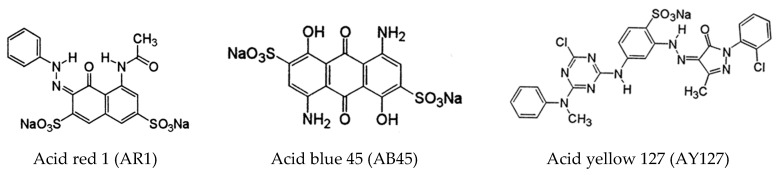
Acid dyes selected in the present study.

The FS along with skins of black drum (*Pogonias comes*) fishes were donated by Big D, Franklin, Louisiana, USA. The selected acid dyes were purchased from Crompton & Knowles Corporation, USA (Scheme 1). All chemicals such as sulfuric acid (Fischer Scientific, Hampton, NH, USA), sodium sulfate (Mallinckrodt, Raleigh, NC, USA), acetone (Sigma-Aldrich, Raleigh, NC, USA) etc. were of analytical grade. 

### 5.2. Phenomenal Description of Gel Formation

**Figure 6:**
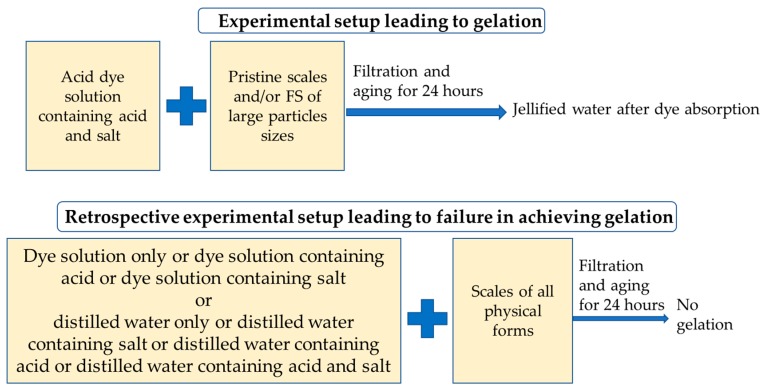
Different experimental designs to watch out gelation phenomena.

**Figure 7:**
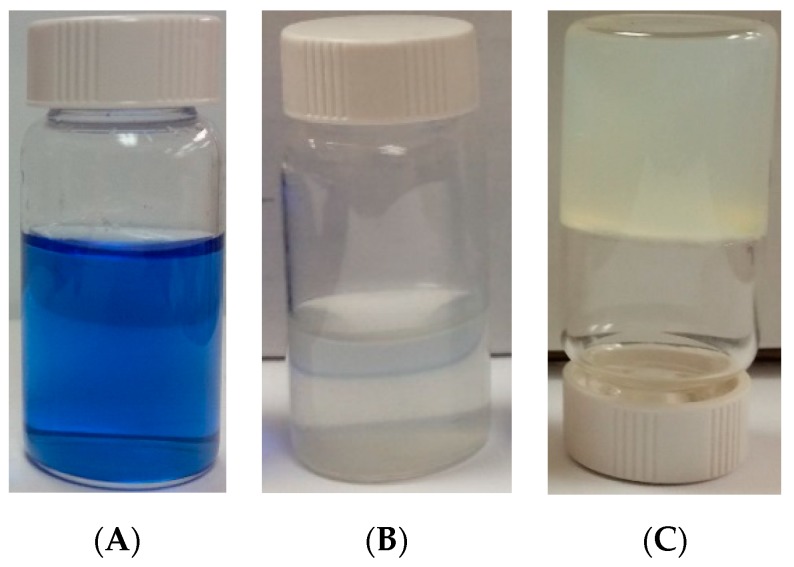
Sample AB45 dye solution (**A**), treated solution (**B**) and jellified solution (**C**).

FS were separated from the skins. They were then washed thoroughly with normal tap water, dried at room temperature, and ground in dry condition using Wiley Mill Model 3 (Thomas Scientific, Swedesboro, NJ, USA). The ground FS were then fractionated using sieves of different mesh sizes (no. 40, 60, 100, and 200). Rest pulverized FS (large) and the whole pristine FS were also used for absorption purpose. Sample dye solutions were prepared following the standard wool dyeing recipe 150 mg/L dye, 62.5 mg/L salt (Na_2_SO_4_), and 7.5 mg/L acid (H_2_SO_4_ [24]. The resulting pH of the solution was 2.5. FS of different sizes were added to the baths containing dye solutions at a ratio of 1:20. The baths were enclosed to prevent them from changing concentration through evaporation. A batch absorption process was applied with continuous stirring at 60 °C for an hour. Finally, the dye-scale mixtures were centrifuged for five minutes at 4000 RPM using IEC (International Equipment Company, Thermo Scientific IEC, Bellport, NY, USA) clinical centrifuge to have clear supernatant. The phenomenal gel formation was examined, by allowing the solutions to age for 24 h at room temperature. In order to understand the underlaying reasons for the gel formation, some retrospective experiments were constructed, but they were unable to form a gel. Figure 6 and Figure 7 illustrate the full set of experiments showing specific conditions of gelation and sample dye solutions at different stages, respectively. The gel content was determined by calculating the residual solids obtained by evaporation (drying) of liquids.

### 5.3. Characterization

#### 5.3.1. FT-IR Analysis

Fourier transform infrared (FT-IR) spectroscopy analysis was conducted to confirm the absorption of dye by FS using Bruker Alpha & Tensor 27 FT-IR & OPUS software (Billerica, MA, USA) with a wavenumber range of 400 and 4000 cm^−1^ [25].

#### 5.3.2. Determination of Dye Removal Efficiency

The dye removal efficiency was evaluated using Equations (1)–(3) and UV-Vis spectroscopy (HP8453, Agilent, Santa Clara, CA, USA) [26]. Here, C_o_ and C_e_ are concentrations of dye in mg/L in wastewaters before and after absorption, X_o_ is the amount of absorbent used in the absorption process in g/L, A is the absorbance, ε is the calibrated molar coefficient in M^−1^ cm^−1^, b is the path length of the cuvette (usually 1cm), c is the concentration in mole/L [4,26,27].

Percentage of dye removal = (C_o_ − C_e_)X100%/C_o_ (1)

q_e_ = (C_o_ − C_e_)/X_o_ (2)

A = εbc(3)

#### 5.3.3. Rheological Analysis

Rheological characterization of the gel obtained after absorption was undertaken by heating and cooling temperature ramp experiments using TA 1000 Rheometer (Thermal Instruments, Trevose, PA, USA). The responses of elastic modulus and viscosity with sweeping temperature were collected. The following parameters have been employed:

- Temperature range: 15–80 °C heating (75–10 °C cooling)
- Temperature step: 5 °C/min
- Frequency: 0.6283 rad/sec
- Oscillation Stress: 0.7958 Pa
- Parallel plate geometry: steel plates 40 mm diameter
- Gap: 300 microns
